# Tracing Human Footprint in Bird Nests: Plastic Occurrence and Drivers in the Long‐Legged Buzzard ( *Buteo rufinus* )

**DOI:** 10.1002/ece3.73948

**Published:** 2026-09-07

**Authors:** Emanuel‐Ștefan Baltag, Vitalie Ajder, Laura‐Elena Topală, Ianula Duță, Viorel Dumitru Gavril

**Affiliations:** ^1^ Marine Biological Research Station “Prof. Dr. Ioan Borcea” “Alexandru Ioan Cuza” University of Iași Agigea Romania; ^2^ ICI RECENT AIR Center, Laboratory of Interdisciplinary Research on the Marine Environment and Marine Terrestrial Atmosphere Al. I. Cuza University of Iasi, Prof. Dr. Ioan Borcea Marine Biological Station Agigea, Constanţa Romania; ^3^ Institute of Ecology and Geography, of the Moldova State University Chisinau Republic of Moldova; ^4^ Faculty of Biology University of Bucharest Bucharest Romania; ^5^ Ovidius University of Constanta, Institute of Doctoral Studies Constanta Romania; ^6^ Institute of Biology Bucharest, Romanian Academy Bucharest Romania

**Keywords:** anthropogenic debris, ecology, macroplastic pollution, raptor nests

## Abstract

Anthropogenic plastic pollution is rapidly becoming embedded in terrestrial ecosystems, yet its long‐term persistence in raptor nesting structures and its transfer across species remain largely overlooked. We investigated the occurrence of macroplastic debris (≥ 20 mm) in nests of the Long‐legged Buzzard ( *Buteo rufinus* ) across its breeding range in Dobrogea, Romania, during the 2025 breeding season. A total of 172 historically recorded nest locations were surveyed, of which 60 were confirmed as actively used by Long‐legged Buzzards and 68 were reused by secondary species, while the remainder were unoccupied, degraded, or had disappeared. Macroplastic materials were recorded in 63.93% of Long‐legged Buzzard nests, a frequency substantially higher than in nests occupied by Common Kestrels ( *Falco tinnunculus* ; 14.89%, *n* = 46) and Eurasian Magpies ( *Pica pica* ; 5.26%, *n* = 19), differences that were statistically significant (*χ* ^2^ = 37.49, df = 2, *p* < 0.001). In all secondary users, plastic debris was confined to the basal nest layer, indicating that materials persisted from original buzzard constructions and were subsequently inherited through interspecific nest reuse. Distance to the nearest settlement was the only statistically significant environmental predictor of plastic abundance (*Z* = −2.878, *p* = 0.004, 95% CI: 0.0002–0.001), with nests located closer to human settlements containing significantly higher numbers of plastic items. These findings reveal that raptor nests can function as long‐term reservoirs of plastic pollution, enabling multi‐seasonal and cross‐species exposure within avian communities sharing the same nesting infrastructure. Our study identifies a plausible and previously overlooked pathway through which anthropogenic waste may accumulate and persist in terrestrial ecosystems, with potential consequences for nestling survival through entanglement and exposure to plastic‐associated contaminants, underscoring the need to integrate plastic pollution monitoring into raptor conservation frameworks.

## Graphical abstract

Long‐legged Buzzard ( *Buteo rufinus* ) nests function as long‐term reservoirs of macroplastics, with plastic material persisting in nest structures and being transferred to other bird species through interspecific nest reuse. This persistence creates an overlooked pathway for the accumulation and spread of plastic pollution in terrestrial ecosystems, with direct implications for avian health, reproductive success, habitat quality, and clear relevance for conservation and ecosystem management.

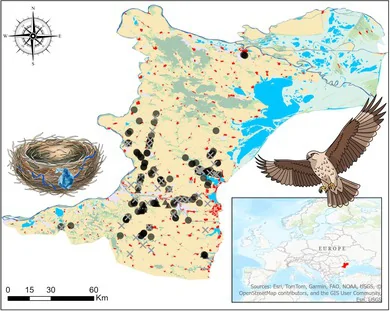

## 1 Introduction

Global waste production reached 2.126 billion tonnes annually in 2020 (UNEP and ISWP 2024), and continues to increase rapidly, with projections indicating that the total volume could double by 2050 (Kaza et al. 2018). Approximately 38% of global waste is disposed of uncontrolled (UNEP and ISWP 2024), resulting in long‐term environmental accumulation, particularly of materials with slow degradation rates. Among these, plastic represents around 10% of solid waste and is considered one of the most pervasive and persistent pollutants, affecting both marine and terrestrial ecosystems (Barnes et al. 2009; Bucci et al. 2020). Unlike biodegradable materials, plastics do not decompose naturally but instead fragment into smaller particles, dispersing globally through the atmosphere, soils, rivers, and oceans (Ter Halle et al. 2016).

Plastic pollution affects wildlife in multiple ways. For birds, the most frequently documented pathways are ingestion and entanglement, yet nest incorporation is increasingly recognized as a third major route of exposure (Battisti et al. 2019). The use of human‐derived materials in nests is not a recent phenomenon—the earliest reference dates back to 1933, when metal wire was recorded in a Pied Crow ( *Corvus albus* ) nest (Warren 1933). Since then, numerous studies have reported birds integrating anthropogenic elements such as plastic filaments, twine, ropes, sheets or cigarette filters into their nests (Suárez‐Rodríguez and Macías Garcia 2014, Battisti et al. 2019; Jagiello et al. 2019, 2022; Harvey et al. 2021; Nguyen et al. 2025). A synthesis of 258 species, mainly seabirds, found that only 1.93% regularly use anthropogenic waste (Battisti et al. 2019); however, more recent studies document substantially higher and alarming values in some species (Janic et al. 2023; Heinze et al. 2025; Nguyen et al. 2025).

The amount and type of plastic in nests vary across species and environments, reflecting both local plastic availability and species‐specific nesting behavior. It is also worth noting that certain avian taxa are known to regurgitate pellets containing anthropogenic materials, which may further influence the local availability and redistribution of plastic debris in the vicinity of nest sites (Wayman et al. 2024). In Osprey ( *Pandion haliaetus* ), the proportion of nests containing baling twine differed significantly between rural, suburban, and urban landscapes (Seacor et al. 2014). In American Crow ( *Corvus brachyrhynchos* ) and White Stork ( *Ciconia ciconia* ), higher plastic loads were reported near agricultural areas and landfills compared to urban sites (Townsend and Barker 2014; Jagiello et al. 2020). Broad patterns indicate that urban and agricultural nest sites contain far more anthropogenic material than natural ones (Potvin et al. 2021). The behavior appears to be partly functional: some birds use plastic as a signal of individual quality (Sergio et al. 2011), when natural materials are scarce (Jagiello et al. 2022), or to reinforce nest structure using durable filaments (Antczak et al. 2010). Anthropogenic materials may also modify nest insulation (Reynolds et al. 2019; Corrales‐Moya et al. 2021). Selective use occurs in some species—for example, Pied Flycatcher ( *Ficedula hypoleuca* ) preferred white plastics in experimental trials (Briggs et al. 2023), Ospreys preferred green materials (Rodríguez et al. 2023), whereas four Paridae species showed no color preference, using materials based on availability (Surgey et al. 2012). Nest material may additionally serve thermoregulatory, antiparasitic or sexual signaling functions, particularly in territorial species (Canal et al. 2016).

Despite these potential benefits, nest incorporation also imposes severe costs. Polypropylene ropes and twine increase entanglement risk for both nestlings and adults (Ryan 2018; Jagiello et al. 2019), often leading to necrosis, limb loss and mortality. Beyond physical entanglement, plastic debris in nests represents only one component of a broader exposure pathway, as accidental ingestion of plastic fragments by nestlings can cause gastrointestinal blockage, endocrine disruption, reduced body condition, and alterations to the gut microbiome, collectively compromising survival and reproductive success (Santos et al. 2021; Tanaka et al. 2020). In White Storks, anthropogenic material occurred in 91% of nests, ropes in 42%, and entanglement injuries in nearly one‐third, affecting 12% of all nestlings (Heinze et al. 2025). Mortality from entanglement has also been reported in Crested Caracara ( *Caracara plancus* ) (Mallet et al. 2020), Osprey (Blem et al. 2002; Restani 2023), White Stork (Janic et al. 2023), American Crow (Townsend and Barker 2014) and Great Gray Shrike ( *Lanius excubitor* ) (Antczak et al. 2010). Exposure is also highly facilitated by poor agricultural waste management, with Europe alone consuming 80 kt of twine annually, much of which is improperly discarded (EIP‐AGRI 2021) and by waste disposal across the inhabited areas (Jagiello et al. 2022; Chen et al. 2024).

While plastic pollution is well‐investigated in marine systems, studies focusing on terrestrial birds remain scarce, despite the fact that most plastic waste is produced inland (Jâms et al. 2020; MacLeod et al. 2021). Research on nest incorporation is still at an early stage (Zduniak et al. 2021; Restani 2023; Espinoza et al. 2024), leaving important gaps in understanding how plastic distribution, land‐use patterns, and proximity to human activity influence nest contamination.

In this context, the present study investigates the prevalence and typology of plastic materials incorporated into the nests of the Long‐legged Buzzard ( *Buteo rufinus* ) in Southeastern Romania (Dobrogea)—one of the country's most biodiverse regions. Furthermore, we assess whether the occurrence of plastic in nests is associated with surrounding land‐use composition and proximity to anthropogenic features, including settlements, waste deposits, and dumpsites. We predict that the number of plastic items incorporated into nests decreases with increasing distance from human infrastructure and in landscapes dominated by natural land‐use types. To our knowledge, this represents the first systematic study of macroplastic incorporation in Long‐legged Buzzard nests globally, providing the first documented evidence of anthropogenic material integration into the nesting structures of this species. Furthermore, this study constitutes the first assessment of plastic debris in bird nests conducted in Romania, contributing a previously absent geographic perspective to the growing body of European literature on plastic pollution in avian nesting environments.

## 2 Materials and Methods

### 2.1 Study Species

The Long‐legged Buzzard is a medium‐sized raptor distributed across arid and semi‐arid regions of northern Africa, southeastern Europe, western and central Asia, extending eastward to China and southward to central India (BirdLife International 2019). In Romania, the species was historically restricted to the Dobrogea region, but in recent decades it has expanded its breeding range toward the north‐eastern part of the country (Baltag 2013). The species primarily hunts in open landscapes, particularly steppe habitats where European ground squirrels ( *Spermophilus citellus* ) and other small mammals are abundant. While nesting was initially recorded mainly on cliffs and occasionally in trees, during the past decade the Long‐legged Buzzard has increasingly adopted high‐voltage electricity pylons as nesting sites, often situated within intensively cultivated agricultural areas (Baltag 2013).

### 2.2 Study Area

**FIGURE 1:**
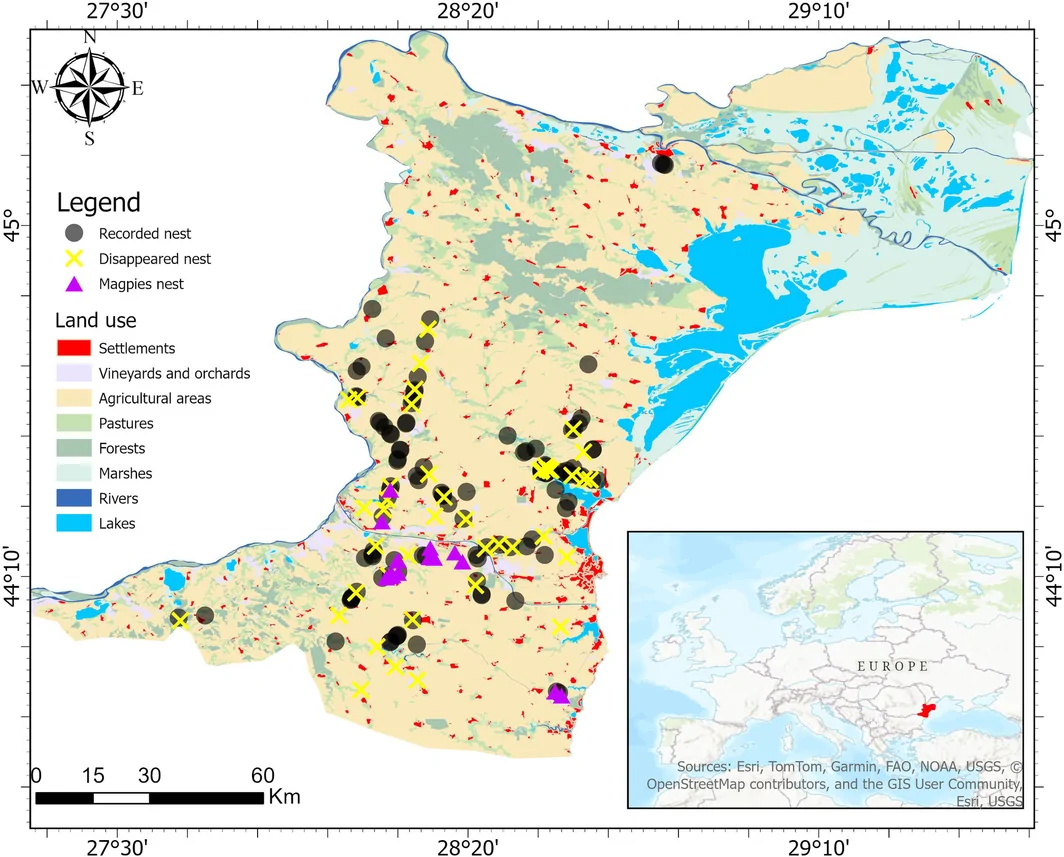
Distribution of surveyed nests within the study area, overlapped with land‐use types based on CLCplus Backbone 2018 data.

The study was conducted in Southeastern Romania, within the Dobrogea Region, with all field evaluations performed during a single breeding season (2025) in order to obtain a consistent snapshot of nest characteristics within the same year (Figure 1). Dobrogea is predominantly a lowland landscape, intersected by hilly formations in the north, including the Măcin Mountains, considered among the oldest mountain systems in Europe. The region has a mean annual temperature of 11.8°C and average annual precipitation of 436.5 mm (Trif and Mihai 2014). Biogeographically, Dobrogea is characterized by steppe and Pontic influences, and is recognized as the richest region in Romania in terms of biodiversity.

### 2.3 Macroplastic Recording

Field data were collected between July and August 2025 during a systematic survey of Long‐legged Buzzard nests in the Dobrogea region. A total of 172 previously known nesting sites, identified from breeding records accumulated over the past 10 years, were revisited to verify their current status. Of these, 44 nests had disappeared prior to the survey and were excluded from all analyses. The remaining 128 nest structures confirmed as physically present were inspected using quadcopter drones (DJI Mini 2 and Autel Robotics Evo2 Dual 640T), allowing a complete overhead view of each nest structure and enabling identification of visible macroplastic fragments (≥ 20 mm) and textile debris. For each nest, multiple aerial photographs (range between 10 and 30 aerial photographs) were taken at varying angles to ensure full spatial coverage and maximize detection of anthropogenic materials. Textile materials were recorded alongside plastic items, as synthetic textiles are known to contain plastic‐derived fibers in their composition and represent one of the most significant sources of microplastic pollution in the environment, with fragmentation of macro‐textile debris into microplastic particles posing a long‐term contamination risk for both the nest environment and the species utilizing these structures.

Physical collection of nest materials for detailed compositional analysis was not attempted for two primary reasons. First, the vast majority of nests are situated on high‐voltage electricity pylons belonging to an international transmission network, rendering direct physical access impossible under standard survey conditions. Second, Long‐legged Buzzards exhibit strong nest‐site fidelity, regularly reusing the same structures across consecutive breeding seasons; physical removal or disturbance of nests could therefore directly compromise future breeding attempts at affected sites.

Several detection limitations inherent to UAV‐based surveys are explicitly acknowledged. First, only plastic items visible on the nest surface could be detected; materials embedded within deeper nest layers are likely to go undetected, and all plastic abundance estimates should therefore be interpreted as conservative minimum counts. Second, nest architecture varies substantially among species: Eurasian Magpie nests are characterized by a domed roof structure that partially obstructs aerial inspection of the nest interior, potentially introducing a downward bias in plastic detection for this species relative to open‐cup nests. This structural difference may affect interspecific comparisons and should be considered when interpreting prevalence differences among species. Third, given that nest volume scales as a cubic function of nest diameter while the visible surface area scales as a quadratic function, larger nests contain proportionally more material within their interior relative to the surface area visible from above. This implies that plastic abundance in large Long‐legged Buzzard nests may be systematically underestimated relative to smaller nests of secondary species, representing an additional source of bias in interspecific comparisons that cannot be fully resolved without physical nest access.

### 2.4 Variables

For each Long‐legged Buzzard nest, macroplastic items were recorded based on photographs obtained using a quadcopter drone. We defined macroplastic as any plastic item larger than 20 mm, following Avery‐Gomm et al. (2018). All identified plastics were classified morphologically into four categories adapted from Espinoza et al. (2024): sheets (e.g., crop‐protector films), hard fragments (broken pieces from larger items), filaments (threads, fibers, strings, ropes), and foamed plastics (e.g., polystyrene). A second categorization considered color, grouped into red/pink, green, blue/purple, black, gray/silver, brown/orange, yellow, and transparent/white (Verlis et al. 2014; Provencher et al. 2017; Grant et al. 2018).

To explore the environmental factors associated with the occurrence of macroplastic in nests, we selected spatial variables considered likely to influence plastic availability. The distance to the nearest urban area was measured, as urban landscapes are major sources of solid waste and are strongly linked to plastic pollution pressure (Forman 2014; Jagiello et al. 2022). We also recorded the distance to the nearest built‐up area (e.g., village, farm), which may act as a more immediate plastic source given the common presence of unmanaged waste near human settlements (Schuyler et al. 2021; Jagiello et al. 2022). Additionally, we quantified the percentage of agricultural land within the species' standard home‐range radius (Maeso et al. 2024), under the assumption that agricultural practices generate plastic residues accessible to raptors while foraging. To assess whether naturally managed landscapes may limit plastic exposure, we further included the proportion of pasture and forest cover within the same spatial buffer in the land‐use analysis.

All spatial variables were extracted using ArcGIS pro (v.3.5.4) by generating nest‐centered buffers of 2 km, corresponding to the estimated home‐range size of Long‐legged Buzzards (6.9 ± 3.5km^2^; Alivizatos et al. 1998). Land‐use percentages were calculated by intersecting buffers with CLCplus Backbone 2018 (2023) layers, while distances to anthropogenic features were computed using the nearest‐distance function (geographic distance in meters).

### 2.5 Statistical Analysis

Patterns of macroplastic occurrence, composition, and abundance in bird nests were examined using a combination of descriptive visualizations and inferential statistical analyses. All analyses were conducted in R (v.4.3.2), primarily using the ggplot2 package for data visualization and the MASS package for generalized linear modeling.

Descriptive graphics were used to summarize interspecific differences in plastic occurrence and characteristics. Proportions of nests containing macroplastic were visualized to compare the prevalence of anthropogenic materials among species occupying Long‐legged Buzzard nesting structures. The composition of plastic types across species was explored using stacked bar representations and tested for interspecific differences using a Chi‐squared test of independence. Color distributions of plastic items were examined in greater detail for the Long‐legged Buzzard, the species with the largest sample size, in order to identify dominant hues and infer potential material sources; the non‐uniform distribution of colors was formally assessed using a Chi‐squared goodness‐of‐fit test against a uniform distribution. The relative contribution of plastic versus textile anthropogenic debris was similarly evaluated using a Chi‐squared goodness‐of‐fit test.

To formally assess the influence of environmental variables on macroplastic abundance, Generalized Linear Models (GLMs) were applied across all 128 nest structures confirmed as physically present during the 2025 breeding season. The number of plastic items per nest was treated as count data and exhibited substantial overdispersion (mean = 1.27, variance = 6.18); therefore, models were fitted using a negative binomial error distribution with a log‐link function, implemented via the glm.nb function in the MASS package in R (v.4.3.2). The goodness‐of‐fit of the final negative binomial model was assessed by computing the Pearson chi‐squared statistic divided by the residual degrees of freedom (Pearson chi^2^/df). Values close to 1.0 indicate adequate model fit (Zuur et al. 2009).

Explanatory variables included distance to the nearest settlement (m) and the surface areas (m^2^) of settlements, agricultural land, pastures, forest, vineyards, wetlands, and water bodies within the estimated home‐range radius of the species. Surface area variables were square‐root transformed prior to modeling to reduce skewness and improve model stability. We acknowledge that these spatial variables represent proxies for plastic availability in the landscape rather than direct measures of waste generation. A degree of uniformity in waste management was assumed across the study area, as all settlements within Dobrogea are subject to the same national waste collection legislation; uncontrolled plastic dispersal was therefore attributed primarily to wind‐mediated transport of lightweight materials and informal disposal practices.

Prior to modeling, predictor variables were examined for multicollinearity using VIF function. Model selection followed a bidirectional stepwise procedure based on the Akaike Information Criterion (AIC), retaining the most parsimonious model explaining variation in plastic abundance. Model fit was evaluated by inspecting residual deviance, dispersion parameters, and the estimated theta parameter of the negative binomial distribution. Statistical significance was assessed at *α* = 0.05, and results are reported as *Z*‐values with associated *p*‐values. All statistical procedures were conducted in R (v.4.3.2) within a reproducible analytical framework.

## 3 Results

### 3.1 Nest Site Occupancy and Species Composition

Using a database of Long‐legged Buzzard breeding sites accumulated over the last decade in Dobrogea, a total of 172 nest locations were surveyed during the 2025 breeding season. Of these, 44 nests could not be located during field surveys and were classified as disappeared, being excluded from all subsequent analyses. Of the remaining 128 nest structures confirmed as present, 60 were recorded as active Long‐legged Buzzard nest structures used during the 2025 breeding season, while the remaining 68 were occupied by secondary species. These included the Common Kestrel ( *Falco tinnunculus* ) at 46 nests (occupied nests, incubating adults or nests with chicks), the Eurasian Hobby ( *Falco subbuteo* ) at three sites, and the Eurasian Magpie ( *Pica pica* ) at 19 sites.

### 3.2 Composition and Characteristics of Macroplastic in Long‐Legged Buzzard Nests

Macroplastic debris was recorded in 37 out of 60 Long‐legged Buzzard nests surveyed, with a total of 143 items identified. Among the plastic material types recorded in Long‐legged Buzzard nests, the distribution was highly non‐uniform (Chi‐squared goodness‐of‐fit test: *χ* ^2^ = 143.74, df = 3, *p* < 0.001), with baling twine dominating overwhelmingly (91 items; 63.6%), followed by plastic bags (44 items; 30.8%), plastic sheets (5 items; 3.5%), and raffia cereal bags (3 items; 2.1%), reflecting the predominant availability of agricultural plastic waste—particularly polypropylene baling twine—in the surrounding landscape.

**FIGURE 2:**
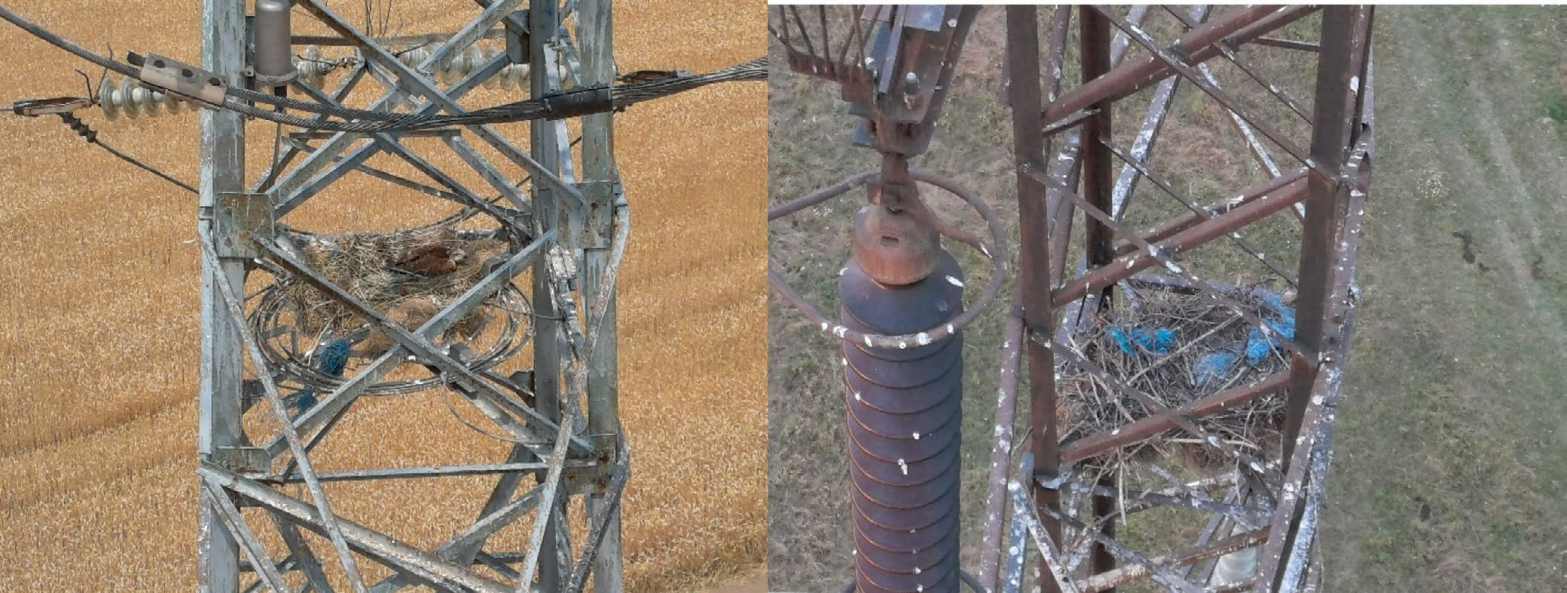
Long‐legged Buzzard nests with blue anthropogenic items (blue polypropylene baler twine, photos by Gavril Viorel).

Blue was the dominant color, recorded in 33 nests (99 items; 68.8%), followed by white in 17 nests (35 items; 24.5%), black in 6 nests (6 items; 4.2%), and brown, red, and green, each recorded in a single nest (1 item each; 0.7%) (Figure 2). The non‐uniform distribution of plastic colors was statistically significant (Chi‐squared goodness‐of‐fit test: *χ* ^2^ = 320, df = 5, *p* < 0.001), confirming that blue items were disproportionately represented.

In addition to plastic, textile materials were recorded in 11 nests, totaling 19 items. Textile debris was predominantly white (11 items in 8 nests), followed by gray (4 items in 4 nests), brown and mauve (2 items each in 2 nests), and single blue and green items recorded in one nest each.

Across all anthropogenic materials recorded in Long‐legged Buzzard nests, plastic items (143 items) substantially outnumbered textile debris (19 items), a difference confirmed as statistically significant (Chi‐squared goodness‐of‐fit test: *χ* ^2^ = 89.82, df = 1, *p* < 0.001), confirming that plastic represents the overwhelmingly dominant category of anthropogenic nest material in this species.

### 3.3 Macroplastic Composition in Secondary Nest Users

In Common Kestrel nests, macroplastic materials were identified in 6 out of 46 nests surveyed, with a total of 18 items recorded. Baling twine was the most frequent material, occurring in five nests (13 items; 72.2%), followed by plastic bags in two nests (3 items; 16.7%) and plastic sheets in one nest (2 items; 11.1%). Blue was the dominant color, recorded in all six plastic‐containing nests (14 items; 70.0%), followed by white in two nests (4 items; 25.0%) and gray in one nest (1 item; 5.0%). Textile material was detected in only one Common Kestrel nest, consisting of two items—one white and one gray.

In Eurasian Magpie nests, plastic was recorded in only one out of 19 nests surveyed, comprising three blue baling twine items, representing 100% of all anthropogenic materials recorded for this species.

### 3.4 Interspecific Differences in Macroplastic Occurrence

**FIGURE 3:**
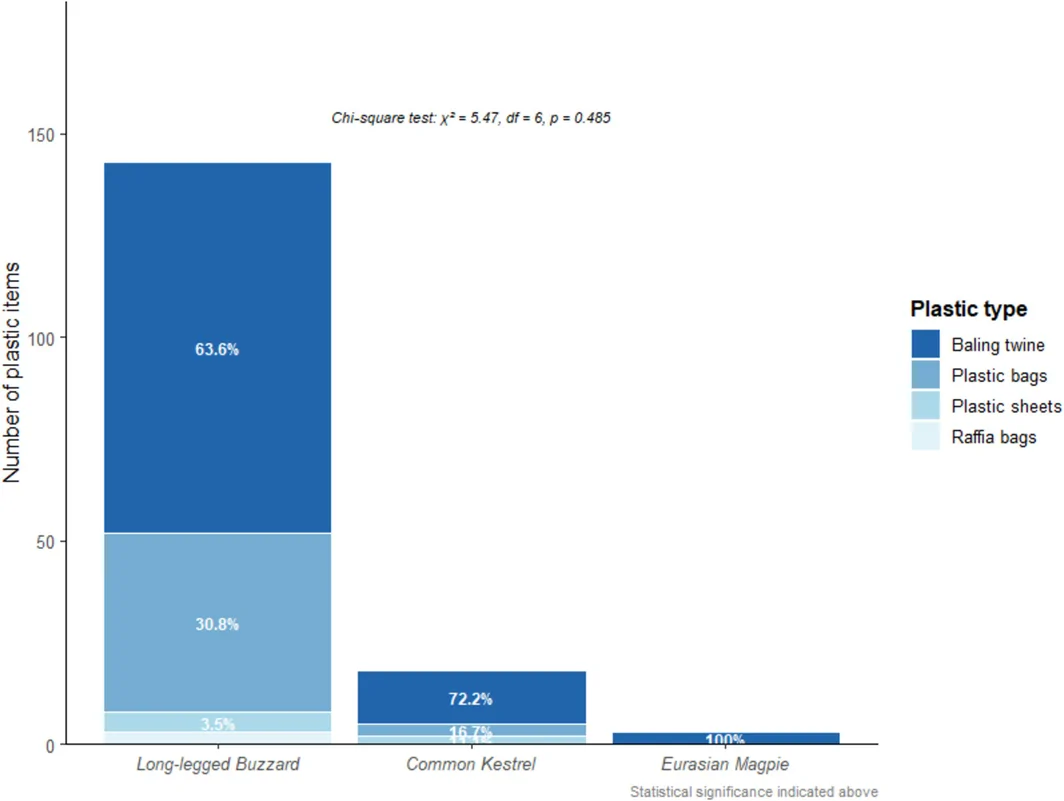
Composition of macroplastic types recorded in nests by species. Stacked bars show the number of items per material category, highlighting the dominance of agricultural baling twine. No statistically significant difference in the distribution of plastic material types was detected among species (Chi‐squared test of independence: *χ* ^2^ = 5.47, df = 6, *p* = 0.485).

The occurrence of macroplastic differed markedly among species groups (Figure 3). Plastic materials were detected in 63.93% of Long‐legged Buzzard nests surveyed (39 out of 60 nests), compared with 14.89% of Common Kestrel nests (7 out of 46 nests) and 5.26% of Magpie nests (1 out of 19 nests). These interspecific differences were statistically significant (Chi‐squared test: *χ* ^2^ = 37.49, df = 2, *p* < 0.001), confirming a strong association between species identity and macroplastic prevalence. Critically, in both Common Kestrel and Magpie nests, plastic items were consistently confined to the basal nest layer—the structural component originally constructed by the Long‐legged Buzzard and subsequently reused by these secondary species.

**FIGURE 4:**
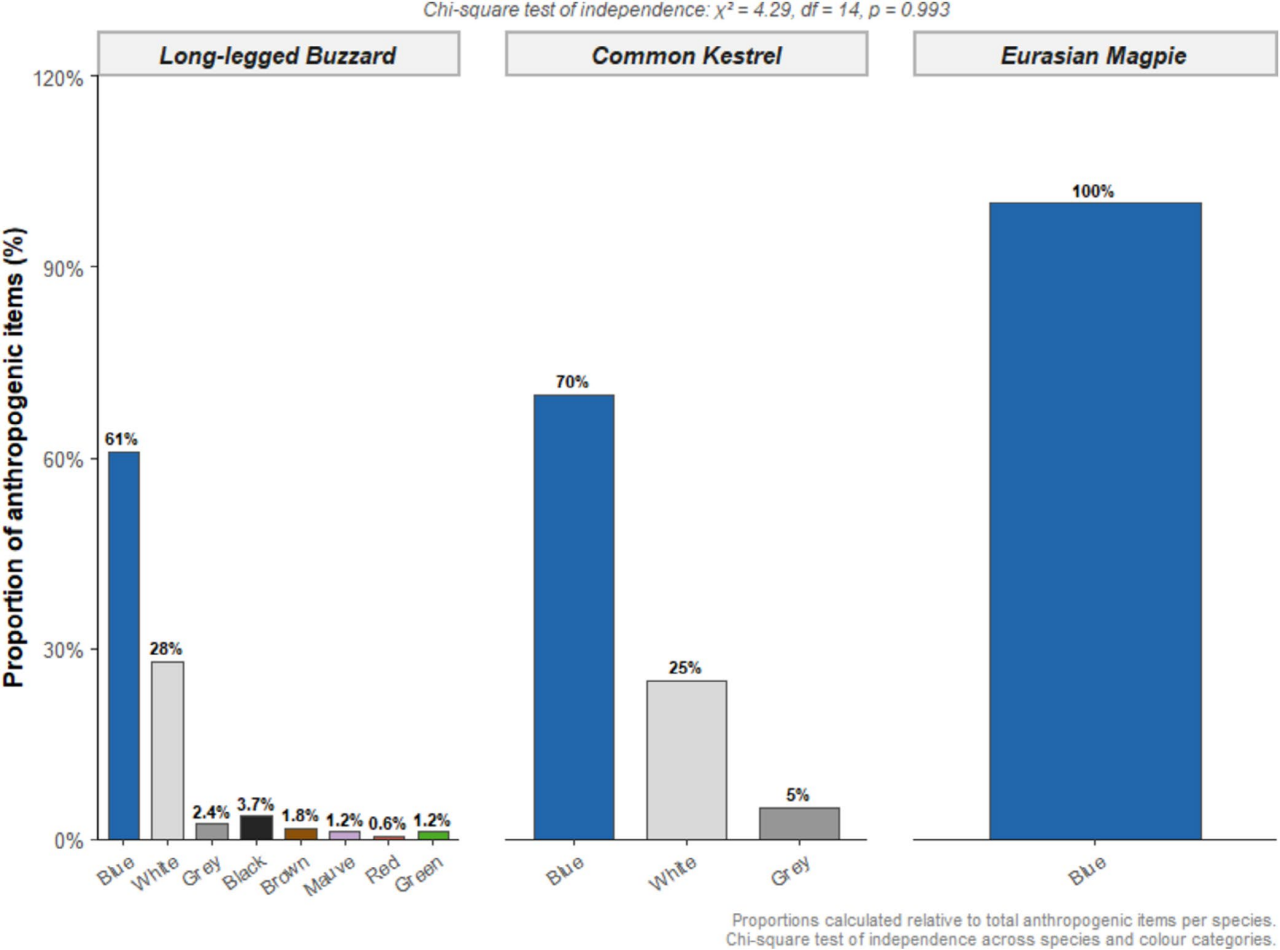
Color distribution of macroplastic items recorded in nests by species. Bars represent the number (or proportion) of items per color category, highlighting the dominance of blue plastics across all species and the broader color diversity observed in Long‐legged Buzzard nests. The non‐uniform distribution of colors was statistically significant (Chi‐squared goodness‐of‐fit test: *χ* ^2^ = 320, df = 5, *p* < 0.001), reflecting the prevalence of blue polypropylene baling twine as the primary source of plastic debris.

The color composition of anthropogenic materials was broadly consistent across all three species, with blue dominating in Long‐legged Buzzard (61.0%), Common Kestrel (70.0%), and Eurasian Magpie nests (100.0%), followed by white as the second most frequent color in both Long‐legged Buzzard (28.0%) and Common Kestrel nests (25.0%) (Figure 4). However, the Chi‐squared test of independence indicated no statistically significant difference in color distribution among species (*χ* ^2^ = 4.29, df = 14, *p* = 0.993), suggesting that the proportional representation of colors was similar across species regardless of the total number of items recorded.

### 3.5 Environmental Predictors of Macroplastic Abundance

A Generalized Linear Model (GLM) with a negative binomial error distribution was applied to assess the influence of environmental variables on macroplastic abundance across all 128 monitored nest structures (mean = 1.27 ± 2.49 items per nest; range: 0–13). The overdispersed nature of the count data (mean = 1.27, variance = 6.18) justified the use of a negative binomial distribution over a standard Poisson model (θ = 0.322, SE = 0.079). Following bidirectional stepwise AIC‐based model selection, forest cover was excluded from the final model (AIC final = 359.19 vs. AIC full = 361.14 vs. AIC null = 381.24), which retained six predictors: distance to the nearest settlement, and the square‐root‐transformed surface areas of settlements, agricultural land, pastures, wetlands, and water bodies (Table 1).

**TABLE 1:** Results of the final Generalized Linear Model (negative binomial distribution, log‐link function) examining the influence of environmental variables on macroplastic abundance across all monitored nest structures (*n* = 128).

Predictor	Estimate	Std. error	*Z*	*p*
Intercept	1.308	1.558	0.839	0.401
Distance to settlement	−0.001	0.0002	−2.878	0.004**
Settlement area	0.001	0.0005	1.940	0.052
Agricultural land	0.001	0.0004	1.844	0.065
Pasture cover	−0.001	0.0003	−1.740	0.082
Wetland area	−0.001	0.001	−1.528	0.126
Water area	0.001	0.001	1.521	0.128

*Note:* Forest cover was excluded during stepwise AIC‐based model selection. AIC null model = 381.24; AIC full model = 361.14; AIC final model = 359.19. Theta (dispersion parameter) = 0.322 ± 0.079; Pearson chi^2^/df = 0.773. Significance codes: **p < 0.01, *p* < 0.1.

Distance to the nearest settlement was the only statistically significant predictor in the final model (estimate = −0.001, SE = 0.0002, *Z* = −2.878, *p* = 0.004, 95% CI: 0.0002–0.001), with nests located closer to human settlements containing significantly higher numbers of plastic items. Settlement surface area (estimate = 0.001, SE = 0.0005, *Z* = 1.940, *p* = 0.052) and agricultural land cover (estimate = 0.001, SE = 0.0004, *Z* = 1.844, *p* = 0.065) showed a marginally non‐significant positive trend, while pasture cover (estimate = −0.001, SE = 0.0003, *Z* = −1.740, *p* = 0.082) exhibited negative but non‐significant associations. Wetland and water surface area did not contribute meaningfully to explaining variation in plastic abundance (*p* > 0.12). Full model results are presented in Table 1.

### 3.6 Evidence for Structural Persistence and Interspecific Transfer of Macroplastics

**FIGURE 5:**
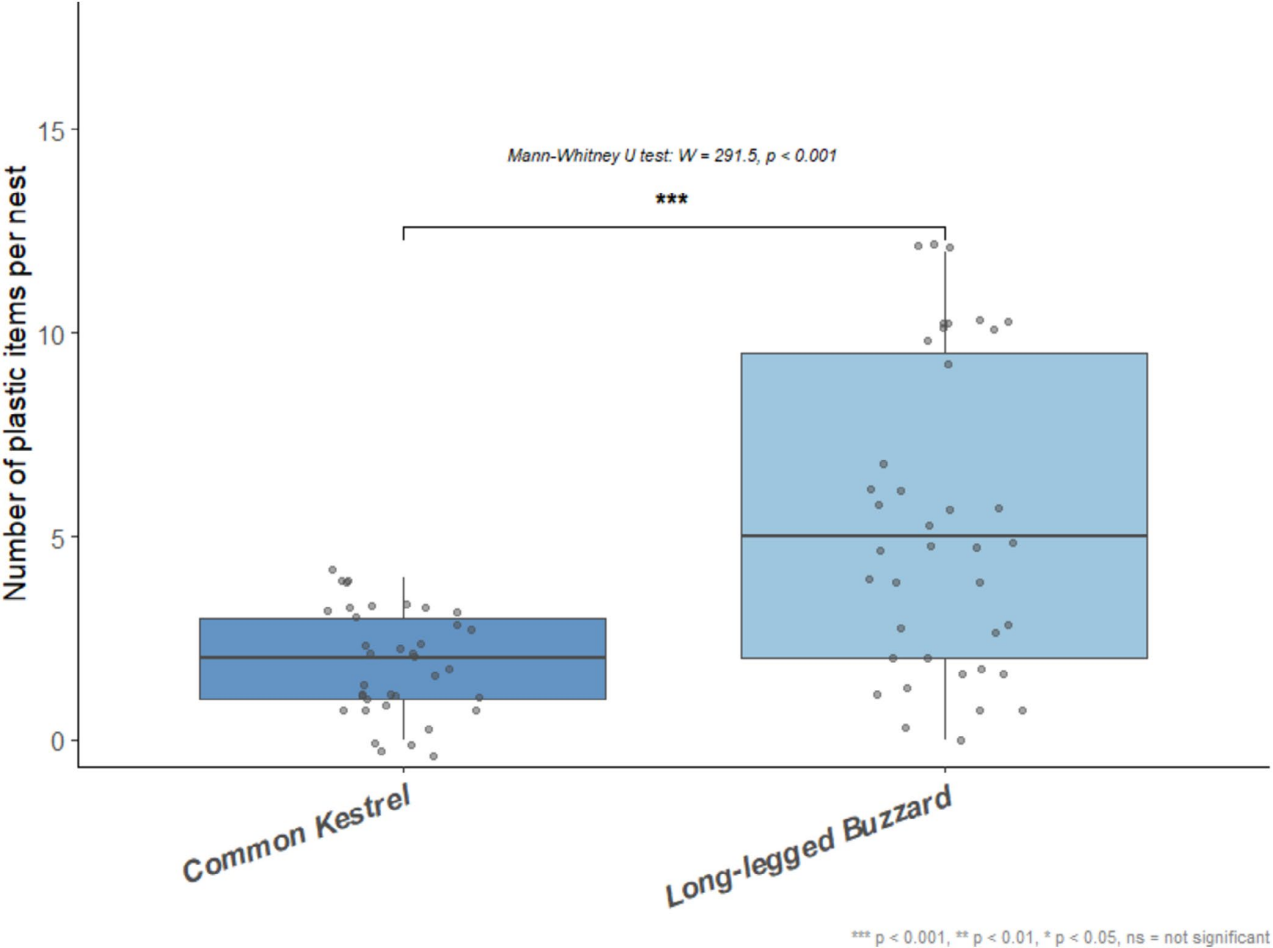
Number of plastic items per nest recorded in Long‐legged Buzzard ( *Buteo rufinus* ) and Common Kestrel ( *Falco tinnunculus* ) nests. Boxes represent the interquartile range (IQR), horizontal lines indicate the median, whiskers extend to 1.5 × IQR, and individual data points are shown as jittered dots. Long‐legged Buzzard nests contained significantly higher plastic loads than Common Kestrel nests (Mann–Whitney *U* test: *W* = 291.5, *p* < 0.001). Eurasian Magpie nests are not shown due to the single plastic‐containing nest recorded for this species.

The prevalence of anthropogenic materials differed markedly among the three species groups. Macroplastic and textile debris were recorded in 63.93% of Long‐legged Buzzard nests (39 out of 60), compared with 14.89% of Common Kestrel nests (7 out of 46) and 5.26% of Magpie nests (1 out of 19), differences that were statistically significant (Chi‐squared test: *χ* ^2^ = 37.49, df = 2, *p* < 0.001). Beyond prevalence, the number of plastic items per nest was also substantially higher in Long‐legged Buzzard nests (median = 5, IQR: approximately 1–9.5, range: 0–13) compared to Common Kestrel nests (median = 2, IQR: approximately 0–3, range: 0–5), a difference confirmed as highly significant by the Mann–Whitney *U* test (*W* = 291.5, *p* < 0.001; Figure 5).

Critically, in both Common Kestrel and Magpie nests, all anthropogenic materials were consistently confined to the basal nest layer—the structural component originally constructed by the Long‐legged Buzzard and subsequently reused by these secondary species—with no evidence of active incorporation by the secondary occupants. This spatial pattern strongly suggests passive inheritance of anthropogenic debris through interspecific nest reuse rather than active material selection.

## 4 Discussion

The present study provides the first systematic assessment of macroplastic and textile incorporation in Long‐legged Buzzard nests and reveals pronounced interspecific differences in the occurrence and composition of anthropogenic materials among raptors and secondary nest users. Nearly two‐thirds of Long‐legged Buzzard nests (63.93%) contained macroplastic items, indicating a very high exposure of this species to human‐derived waste. In contrast, plastic was detected in only 14.89% of Common Kestrel nests and 5.26% of Magpie nests, and in both cases exclusively within the basal nest layers. This pattern strongly suggests passive inheritance of materials originally incorporated by Long‐legged Buzzards rather than active selection by secondary occupants.

Comparable levels of plastic incorporation have been reported in other raptor species, although with pronounced regional and ecological variability. In southern Spain, for instance, 77% of Black Kite ( *Milvus migrans* ) nests were decorated with plastic materials, predominantly white items (Sergio et al. 2011), while nearly half of Osprey ( *Pandion haliaetus* ) nests across the Yellowstone River basin contained plastic baling twine (Restani 2023). In contrast, a study on Black Storks ( *Ciconia nigra* ) in Poland documented plastic in only 26% of nests, with occurrence increasing closer to forest edges (Janic et al. 2023). Collectively, these findings indicate that plastic incorporation into nests is highly context‐dependent, reflecting species‐specific nesting behavior and local land‐use characteristics. In the present study, Long‐legged Buzzards exhibited a marked predominance of blue and white plastics, which together accounted for 93.75% of all macroplastic items recorded in nests. While white plastic was also the dominant color reported in Black Kite nests in Spain (Sergio et al. 2011), it ranked second in Long‐legged Buzzard nests, where blue items were most frequent, representing 68.75% of the total. The ecological or behavioral mechanisms underlying this color pattern remain unclear and warrant further investigation in future studies.

In the present study, baling twine emerged as the dominant plastic material across all species, accounting for the majority of recorded items. Its prevalence reflects the extensive use of polypropylene twine in agricultural practices and its persistence in open landscapes. The dominance of blue‐colored plastic, which represented the vast majority of items, further supports an agricultural origin, as blue twine is widely used in crop management and hay baling. These patterns are consistent with previous studies identifying agricultural waste as a primary source of nest‐incorporated plastics in raptors and other large birds nesting in farmland‐dominated environments (Antczak et al. 2010; Seacor et al. 2014; Jagiello et al. 2019). The recurrent presence of plastic bags and sheets additionally indicates dispersal from nearby settlements and agricultural infrastructure, highlighting multiple pathways through which plastic becomes accessible at nest sites.

The results of the Generalized Linear Model provide quantitative support for the hypothesis that human proximity is a primary driver of macroplastic incorporation in Long‐legged Buzzard nests. The significant positive effect of distance to the nearest settlement (*Z* = −2.878, *p* = 0.004) indicates that nests situated closer to human settlements consistently accumulated higher numbers of plastic items, a pattern consistent with findings reported for other raptor and waterbird species nesting in human‐modified landscapes (Jagiello et al. 2019; Kwieciński et al. 2024; Lasota et al. 2024). This relationship likely reflects the greater availability and dispersal of plastic waste in the immediate vicinity of settlements, where inadequate waste management and wind‐mediated transport facilitate the deposition of anthropogenic materials in the surrounding environment.

Interestingly, while settlement surface area (*Z* = 1.940, *p* = 0.052) and agricultural land cover (*Z* = 1.844, *p* = 0.065) showed a marginally non‐significant positive trend, pasture extent exhibited negative, albeit non‐significant, associations with plastic abundance (*Z* = −1.740, *p* = 0.082). Both agricultural areas and peri‐urban environments represent relevant sources of macroplastic—the former through the use of specific agricultural materials such as baling twine, mulching films, and irrigation equipment, and the latter through the convergence of multiple diffuse waste streams including packaging, construction debris, and household refuse. The absence of a significant effect of broader land‐use composition variables—including forest, wetland, and water cover—further reinforces the conclusion that it is the proximity to point sources of anthropogenic waste, rather than the overall landscape matrix, that most strongly determines plastic exposure at individual nest sites.

These findings have important implications for understanding the spatial distribution of plastic pollution risk in raptor populations. Given that Long‐legged Buzzards in Dobrogea predominantly nest on high‐voltage electricity pylons distributed across an agricultural matrix, variation in plastic load among nests is likely to reflect fine‐scale differences in the proximity of individual pylons to human infrastructure rather than broad habitat preferences. The ecological implications of plastic incorporation are substantial. Long‐legged Buzzard chicks feed directly from prey items delivered to the nest floor, increasing the likelihood of accidental ingestion of plastic fragments. Similar mechanisms have been documented in White Storks, where plastic ingestion led to choking, gastrointestinal blockage, and starvation due to gut occlusion (Henry et al. 2011; Jagiello et al. 2018). In addition, plastic strings and twine pose a particularly severe risk through entanglement, which can result in limb injuries, amputations, and mortality of nestlings, as documented across multiple bird species (Votier et al. 2011; Townsend and Barker 2014; Jagiello et al. 2018; Janic et al. 2023; Heinze et al. 2025). Beyond immediate physical hazards, the long‐term physiological consequences of plastic exposure in raptors may be equally significant. As macroplastic debris fragments over time into smaller particles within the nest environment, nestlings are increasingly exposed to microplastic ingestion, which has been associated with biomagnification of persistent organic pollutants and plastic‐associated chemical additives along food chains, immune system impairment, endocrine disruption, and altered hormonal regulation—all of which may compromise growth, survival, and long‐term reproductive success in affected individuals (Tanaka et al. 2020; Santos et al. 2021).

While the present study indicates that plastic incorporation in Long‐legged Buzzard nests is largely passive, evidence from other taxa suggests that active selection may occur in certain species. European Serins ( *Serinus serinus* ) and Eurasian Magpies, for example, preferentially select filamentous plastics despite their relatively low environmental availability, with European Serins incorporating such materials almost exclusively among anthropogenic nest components (Espinoza et al. 2024). Kentish Plovers ( *Charadrius alexandrinus* ) have also been shown to select lighter‐colored nest materials, likely to reduce thermal stress (Gómez et al. 2019). Nest decoration may additionally serve behavioral functions, such as delaying egg removal by predators (Husby and Slagsvold 2025), although conspicuous materials can also increase nest detectability (Canal et al. 2016), illustrating the complex trade‐offs involved.

The substantially higher occurrence and quantity of plastic in Long‐legged Buzzard nests compared to those of Common Kestrels and Magpies is likely linked to differences in nest size, construction behavior, and nest‐site fidelity. Long‐legged Buzzards build large, durable nests that are reused over multiple breeding seasons, allowing materials to accumulate over time. In contrast, Common Kestrels typically use smaller or simpler nesting structures with minimal modification, while Magpies frequently renew or reconstruct nests, potentially limiting long‐term accumulation of anthropogenic debris.

Finally, the presence of textile materials in a subset of Long‐legged Buzzard nests highlights an additional and often overlooked category of anthropogenic debris. Although less abundant than plastics, textiles may pose similar risks, including entanglement and altered nest microclimate. Given the high confidence association between plastic abundance and proximity to human settlements, and the dominance of agricultural materials such as baling twine, our findings underscore the need for improved waste management practices in agricultural landscapes.

Overall, these results demonstrate that macroplastic incorporation into bird nests is primarily driven by land‐use context and material availability rather than active selection alone. Through their large, persistent nests and strong association with open, human‐modified landscapes, Long‐legged Buzzards emerge as effective bioindicators of terrestrial plastic pollution, complementing the predominantly aquatic focus of existing plastic pollution research.

These findings have direct implications for conservation management. Given the overwhelming dominance of blue polypropylene baling twine as the primary plastic source, a critical unresolved question is whether Long‐legged Buzzards actively select blue‐colored materials or incorporate them passively due to their high environmental availability. We therefore propose that controlled color preference experiments should be prioritized as a first step, as their outcomes would directly inform the most appropriate management pathway. If an active preference for blue is confirmed, the most targeted and immediately actionable intervention would be to promote the adoption of baling twine in alternative colors less visually attractive to raptors—a relatively low‐cost modification requiring minimal changes to existing agricultural practices. If no color preference is demonstrated, the priority should shift toward replacing conventional polypropylene twine with biodegradable alternatives, thereby addressing the persistence of plastic debris in the environment regardless of bird behavior. In both scenarios, systematic collection of used baling twine and agricultural plastic waste at farm level, combined with improved general anthropogenic waste management in the vicinity of active nest sites, are essential parallel measures.

## Author Contributions

**Emanuel‐Ștefan Baltag:** conceptualization (lead), data curation (lead), formal analysis (lead), funding acquisition (lead), investigation (lead), methodology (lead), project administration (lead), resources (lead), software (lead), supervision (lead), validation (lead), visualization (lead), writing – original draft (lead). **Vitalie Ajder:** investigation (equal), resources (equal), writing – review and editing (equal). **Laura‐Elena Topală:** writing – review and editing (equal). **Ianula Duță:** writing – review and editing (equal). **Viorel Dumitru Gavril:** investigation (equal), resources (equal), writing – review and editing (equal).

## Funding

This work was supported by Ministerul Cercetării, Inovării şi Digitalizării, CNCS—UEFISCDI, project number PN‐IV‐P2‐2.1‐TE‐2023‐0897 and Institute of Biology Bucharest of Romanian Academy, project no. RO1567‐IBB09/2026.

## Conflicts of Interest

The authors declare no conflicts of interest.

## Supporting information


**Data S1:** ece373948‐sup‐0001‐supinfo.xlsx.

## Data Availability

The data supporting the findings of this study are available in the Supporting Information.
